# Clinical Outcomes of Long Head Biceps Tendinitis Treatment by a Semitenodesis Technique

**DOI:** 10.7759/cureus.31430

**Published:** 2022-11-12

**Authors:** Ahmed A Alghamdi, Raad M. M Althaqafi, Yasser H Babaier, Mohamed S Singer, Sara Assiri, Bakar Aljohani, Faisal A Alghamdi, Ahmed Abdel Badie

**Affiliations:** 1 Orthopedic Surgery, College of Medicine, Al-Baha University, Al Baha, SAU; 2 Orthopedic Surgery, King Abdulaziz Specialist Hospital, Taif, SAU; 3 Orthopedics, Alhada Armed Forces Hospital, Taif, SAU; 4 Orthopedics, Al Hada Military Hospital, Taif, SAU; 5 Otolaryngology - Head and Neck Surgery, King Faisal Hospital, Taif, SAU; 6 Neuroradiology, Al Hada Military Hospital, Taif, SAU; 7 Orthopedics, College of Medicine, Taif University, Taif, SAU; 8 Orthopedics, Suez Canal University, Suez, EGY

**Keywords:** semitenodesis technique, long head biceps, semitenodesis, tenodesis, technique, new, lhb

## Abstract

Introduction

Long head biceps (LHB) tendon pathology results in anterior shoulder pain, affecting activities requiring overhead movement and forward flexion. Current surgical options for those in whom conservative management failed include tenotomy and tenodesis, and both have considerable success rates and various complications. Herein, we present a novel technique using tenotomy with autotenodesis of the LHB.

Methods

Patients with isolated LHB tendinopathy and for whom the six-month conservative treatment failed were included in our study. Our semitenodesis technique was performed from May 2015 to May 2021. All patients underwent postoperative rehabilitation and were followed in the clinic to document the visual analog scale (VAS) score, constant functional score, supination and flexion power, and postoperative satisfaction score. We used IBM SPSS Statistics for Windows version 20.0 (IBM Corp., Armonk, NY) to analyze our data.

Results

The study included 26 patients with a mean age of 50 ± 4.3 years and a male predominance. Following our technique, the postoperative VAS score improved significantly from 8.8 to 3 within three months and decreased to 0.4 during the final follow-up. The shoulder average constant score improved significantly from 45 ± 4 to 79 ± 5, in addition to a high postoperative mean satisfaction score. Only one patient had a Popeye sign deformity, making an incidence percentage of 3.8% with our technique.

Conclusions

We conducted this study to assess the outcomes of our novel technique using tenotomy with autotenodesis of the LHB compared to traditional techniques such as tenotomy and tenodesis. Our novel technique showed an improvement in pain score superior to patients who underwent tenodesis and tenotomy three months postoperatively. Furthermore, our technique yielded lower postoperative complications than traditional techniques. Our patients also scored a high mean of postoperative satisfaction. Therefore, our technique is a promising treatment option, proving its superiority over tenotomy and tenodesis in treating isolated LHB tendonitis.

## Introduction

Anterior shoulder pain originating from long head biceps (LHB) tendon pathology can be incapacitating and cause impairment of daily living activities [1]. LHB tendinopathy pain is usually aggravated during overhead movement and forward flexion, limiting the active range of motion [2]. The pathology of LHB includes acute traumatic tears, chronic tendinitis, degenerative changes, and overuse and misuse injuries. Such pathology results in an inflamed tendon with degeneration and tenderness to palpation or stretch during shoulder motion [3]. Such tendinopathy could be isolated but is commonly associated with rotator cuff (RC) pathology [1].

Nonsurgical treatment occurs in a considerable percentage of patients with LHB tendinopathy and warrants surgical intervention. Currently available surgical solutions include tenotomy and tenodesis [1,4]. Despite the considerable success of these two surgical techniques in alleviating tendinopathy-associated pain, each is associated with various complications and undesirable outcomes.

The LHB tenotomy prevents traction pressure on the LHB's inflamed, teared, or degenerated tendon, thus relieving its emanating pain [5]. However, tenotomy can cause LHB distal migration [6]. LHB distal migration varies such that it can lead to a cosmetic deformity (the Popeye shape). These variable degrees of deformities are rare and age-dependent and are more commonly found among older patients than younger ones [7]. Other post-tenotomy complications include spasms and discomfort in the biceps muscle [5,8] and reduced elbow flexion and external rotation power compared to the normal contralateral arm [9,10]. Elbow strength limitation is somewhat important among younger patients who live active, laborious lifestyles but not older patients [7].

Tenodesis removes the pathological tendon part, abolishes proximal tendon angulation, and gives the LHB a new fixation anchor in the proximal humerus, thus keeping the length-tension relationship of the LHB musculotendinous unit [11,12]. Arthroscopic LHB tenodesis performed at the articular margin results in a modest percentage of surgical revision, a low rate of remaining pain, and significant progress in the outcomes of the target shoulder [12]. However, tenodesis is a rather complicated surgical procedure that requires a postoperative fixation and long rehabilitation [7]. While reliable, tenodesis is associated with problems such as failure of fixation and biceps pain, which may present at the implant-bone-tendon contact [13,14], with common failure of the interface between the implant and the tendon [7]. Furthermore, poor patient satisfaction is caused by LHB length-tension mismatch, infection, hematoma, neurologic injuries, vascular injuries, iatrogenic fractures, and reflex sympathetic dystrophy. Such complications have been reported but are less common in biceps tenodesis [15,16]. In a trial to avoid and decrease complications associated with tenodesis and tenotomy, we used tenotomy with a semitenodesis variation of the LHB autotenodesis technique. The present study aims to present the outcomes of our technique.

## Materials and methods

Patient selection and data collection

The study included all patients with symptomatic LHB tendinopathy who had arthroscopic LHB semitenodesis for whom six months of conservative treatment failed. The study excluded patients with concomitant RC tears requiring repair, patients with inflammatory arthropathy, or adhesive capsulitis. Data were collected prospectively and retrospectively reviewed between May 2015 and May 2021. A total of 26 patients were included in the current study. All enrolled patients underwent a conservative treatment consisting of physical therapy and nonsteroidal anti-inflammatory drugs for at least six months before surgery.

Ethical considerations

The study was approved by the Research Ethics Committee at Al-Hada Armed Forces Hospital (reference number: 19200). All patients in the study provided written informed consent following an explanation of the procedures and the study.

Operative technique

**Figure 1:**
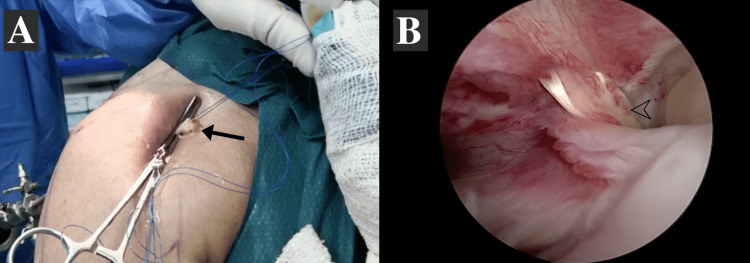
Delivering of long head biceps (LHB) tendon through the portal. (A) Image of the LHB tendon delivered through the anterolateral portal (arrow) being sutured on itself by a nonabsorbable suture. (B) Interarticular view showing the LHB tendon delivered through the portal (arrowhead).

The standard beach chair position was used in all patients after the induction of general anesthesia. The operating arms were sterilized and draped according to the hospital protocol, and a single dose of preoperative antibiotic was administered 30 minutes before surgery. Standard posterior portals were created, and diagnostic shoulder arthroscopy was performed to ensure LHB tendon pathology. Through the anterolateral portal, the LHB was held by a Kocher forceps, and an electrical cautery device was introduced from the same portal to tenotomize the tendon at insertion. Next, the LHB tendon was delivered through the portal with the elbow in a flexion position (Figure 1). The proximal 1 cm of LHB was flipped and sutured on itself using a nonabsorbable suture (Ethibond Suture 5, Ethicon, Inc., Raritan, NJ). Finally, the tendon was returned intraarticularly by removing the Kocher with elbow extension. The final arthroscopic examination showed that the LHB tendon was hanging at the pulley or proximal bicipital groove.

Postoperative rehabilitation

The patients' arms were placed in an arm sling for a few days for rest; active and passive elbow and shoulder motion was allowed. The patients were followed in the clinic, and the visual analog scale (VAS) score was reported three months after surgery and at the final follow-up. The constant functional score and supination and flexion power were also reported at the final follow-up. Power was measured three times using a dynamometer with a one-minute rest, and we recorded the average reading as a percentage compared to the normal contralateral side. The satisfaction score was recorded as 0 to 1s0, with 10 being the most satisfied. We compared the preoperative and postoperative constant functional scores, the incidence of Popeye deformity, and the pain on the VAS of our semitenodesis technique with that of tenotomy and tenodesis from previous studies to evaluate the technique's efficiency [17-20].

Statistical analysis

We used IBM SPSS Statistics for Windows version 20.0 (IBM Corp., Armonk, NY) to analyze the data. The qualitative data variables were expressed as frequencies and percentages, while the quantitative variables were expressed as means and standard deviations. An independent sample t-test was used to identify significant differences between variables, and p < 0.05 was considered statistically significant.

## Results

A total of 26 patients were included in the study. The patient population had a mean age of 50 ± 4.3 years, with 21 men (80.8%) and five women (19.2%). Table 1 presents the sociodemographic data. Most patients had right shoulder LHB surgery (88.5%), while only three had left shoulder surgery (11.5%). The indications for LHB semitenodesis were degenerative tendinopathy and tears (n = 20), superior labrum from anterior to posterior (SLAP) lesion (n = 4), and instability with ruptured pulley with partial cuff tear (n = 2). The most common comorbidity among our cohort of patients was type 2 diabetes (n = 7; 26.9%) followed by hypertension (n = 3; 11.5%) and hyperthyroidism (n = 1; 3.8%). Patients experienced a mean of nine months (range: seven months to one year) of symptoms before undergoing surgical intervention.

**Table 1:** Sociodemographic data (N = 26) SD, standard deviation; SLAP, superior labrum from anterior to posterior.

Baseline characteristics	Mean ± SD	N (%)
Mean age (years)	50 ± 4.31	
Mean body mass index (kg/m^2^)	22.1 ± 2.73	
Male		21 (80.8%)
Female		5 (19.2%)
Smokers		6 (23.1%)
History of smoking		2 (7.7%)
Type 2 diabetes		7 (26.9%)
Hypertension		3 (11.5%)
Hyperthyroidism		1 (3.8%)
Right side is affected		23 (88.5%)
Left side is affected		3 (11.5%)
Degenerative tendinopathy and tears		20 (76.9%)
SLAP lesion		4 (15.4%)
Instability with ruptured pulley with partial cuff tear		2 (7.7%)

Table 2 presents the functional preoperative and postoperative results. The pain score improved significantly from 8.8 ± 0.4 preoperatively to 3 ± 1.6 three months postoperatively (p < 0.001) and 0.4 ± 0.7 (p < 0.001) at the last postoperative follow-up. The mean shoulder constant score improved significantly from 45 ± 4 to 79 ± 5 postoperatively at the final follow-up (p < 0.001). The average elbow flexion and supination value decreased to 94% and 91% on the contralateral side, respectively. Preoperative flexion and supination data were not available. The mean postoperative patient satisfaction score was 9.2 ± 1.6. Only one patient had Popeye sign deformity postoperatively (3.8%), and no neurovascular complications were reported.

**Table 2:** Functional preoperative and postoperative results ^a ^Pain measured using the visual analog pain scale, with 0 as no pain and 10 as most extreme pain. ^b ^Satisfaction scale of 1 being most unsatisfied and 10 being very satisfied.

Preoperative and postoperative factors	Mean ± SD	N (%)
Received conservative therapy		26 (100%)
Mean duration of symptoms appearance until surgical intervention (months)	9.15 ± 1.43	
Preoperative pain^a^	9.80 ± 0.40	
Pain at one month postoperative^a^	4.15 ± 1.64	
Pain at three months postoperative^a^	0.40 ± 0.70	
Preoperative shoulder flexion (degrees)	150.19 ± 8.30	
Three months postoperative shoulder flexion (degrees)	154.03 ± 7.21	
Postoperative shoulder flexion improvement (degrees)	3.8 ± 4.0	
Postoperative elbow flexion (power grade)	4.9 ± 0.39	
Postoperative Popeye sign		1 (3.8%)
Postoperative patients’ satisfaction^b^	9.6 ± 1.44	

## Discussion

Continuous improvement of surgical procedures to minimize operation-associated complications, accelerate recovery, and reduce patient pain is a must for all health practitioners, especially surgeons. In the current study, we implemented a new surgical technique (semitenodesis) to minimize traditional surgical techniques' well-known complications. The failure of nonsurgical medical management of LHB tendonitis necessitates surgical intervention using tenotomy or tenodesis, which are well-known, established techniques whose superiority is debated [21,22]. The VAS pain score in patients who underwent the novel arthroscopic semitenodesis technique in this study showed a 58% improvement from 9.8 to 4.15 one month after the operation and a 96% improvement from 9.8 to 0.4 three months after the operation. The 58% improvement in pain score achieved in this study at four weeks is faster than the previously reported 57% improvement attained at six weeks in patients who underwent tenodesis [23]. Additionally, the near absence of pain (96% improvement) was achieved at three months postoperatively in our study, while the same postoperative time resulted in only 78% improvement in patients who underwent tenodesis [23]. The VAS pain score (96% improvement) achieved three months after semitenodesis in our patients is superior to the VAS pain score reduction of approximately 30% after tenodesis and 47% after tenotomy at three months postoperatively [17].

The enlarged bulging of the distal part of the biceps muscle is called Popeye deformity, which commonly occurs after biceps tenotomy for surgical treatment. In a 24-month follow-up study, Popeye incidence was 33% after tenotomy and 9.5% after tenodesis [24]. Another study showed a similar trend; Popeye deformity was reported to be 25% after tenotomy and 7% after tenodesis [17]. Although tenodesis resulted in a remarkable decrease in Popeye deformity formation compared to tenotomy, the procedure is more challenging to perform and is associated with the risk of implant complications and a prolonged immobilization time during rehabilitation [25]. Compared to these previously reported Popeye incidences, our semitenodesis technique showed superiority with only one case of Popeye deformity among 26 patients (3.8% incidence). This superiority of semitenodesis is attributed to this new operation technique's efficiency because the flipped tendon's migration was stopped in the pulley or transverse humeral ligament, preventing such a Popeye deformity.

Semitenodesis yielded a constant improvement in the functional score (34 points), superior to that of tenotomy (15 points) and tenodesis (21 points; Table 3) [17-20]. Postoperative patient satisfaction after the semitenodesis surgical technique was highly positive, with a mean of 9.6 ± 1.44 (10 being very satisfied). Tenotomy yielded a postoperative patient satisfaction of 75%, while tenodesis yielded an 88% postoperative satisfaction score [20]. Further emphasizing the superiority of semitenodesis, the novel technique yielded a 96% patient satisfaction score. However, the improvement was recorded at three months postoperatively compared to 30 months for tenotomy and tenodesis [20].

**Table 3:** Semitenodesis compared to tenotomy and tenodesis

Outcomes	Semitenodesis	Tenotomy	Tenodesis
Mean preoperative pain	9.80 ± 0.40	7.5 [17]	6.2 [17]
Mean three months postoperative pain	0.40 ± 0.70	4 [17]	4.4 [17]
Mean preoperative shoulder flexion (degrees)	150.19 ± 8.30	156.6 [18]	143.6 [18]
Mean three months postoperative shoulder flexion (degrees)	154.03 ± 7.21	156.4 [18]	154.1 [18]
Postoperative improvement in shoulder flexion (degrees)	3.8 ± 4.0	0.2	10.5
Postoperative elbow flexion (power grade)	91	0.93 ± 0.19 [19]	0.92 ± 0.15 [19]
Preoperative constant functional score	45 ± 4	58.0 ± 14.2 [19]	53.6 ± 13.4 [19]
Postoperative constant functional score	79 ± 5	73.8 ± 11.2 [19]	74.8 ± 11.9 [19]
Postoperative improvement in constant functional score	34	15	21
Postoperative Popeye sign incidence	3.8%	24.7% [19]	7.5% [19]
Postoperative patient satisfaction	96%	75% [20]	88% [20]

Furthermore, one study reported postoperative improvement in shoulder flexion was 0.2 degrees for the tenotomy and 10.2 degrees for tenodesis [18], while semitenodesis achieved a midway improvement of 3.8 degrees (Table 3). However, the postoperative improvement recorded time was only three months in the current study compared to 24 and 26 months for tenotomy and tenodesis [18].

Our study had a few important limitations. Our study population included a relatively small number of cases, and we did not directly compare outcomes with other procedures, such as tenotomy or tenodesis, in a controlled setting. Further comparative studies and studies of the technique are recommended in patients with concomitant RC surgery.

## Conclusions

This study aimed to present the outcomes of a novel tenotomy using a semitenodesis variation of the LHB autotenodesis technique. The simple procedure for managing LHB pathology yielded excellent functional results, great patient satisfaction, and very few complications. This new technique showed an improvement in pain and constant functional scores compared to tenodesis and tenotomy three months postoperatively. Given the improvement in pain score, the constant functional score, reduced Popeye incidence, high patient satisfaction rate, and most importantly, the short time in which these were achieved, semitenodesis appears superior to tenotomy and tenodesis and should be considered by shoulder surgeons as a promising new treatment of isolated LHB tendinitis.
